# Hepatobiliary PET with [^68^Ga]Ga-BP-IDA – preclinical evaluation and its translational potential for liver function monitoring

**DOI:** 10.1186/s13550-025-01327-2

**Published:** 2025-10-02

**Authors:** Julia Greiser, Robert Drescher, Marta Pomraenke, Mitali Sonawane, Olga Perkas, Christian Kuehnel, Thomas Scholz, Sebastian Groeber, Thomas Weisheit, Adrian Press, Thomas Winkens, Nathalie Viohl, Anke Werner, Michael Bauer, Martin Freesmeyer

**Affiliations:** 1 Working Group for Translational Nuclear Medicine and Radiopharmacy, Clinic of Nuclear Medicine, Jena University Hospital, Am Klinikum 1, 07747 Jena, Germany; 2 Clinic of Nuclear Medicine, Jena University Hospital, Am Klinikum 1, 07747 Jena, Germany; 3 Department of Anaesthesiology and Intensive Care Medicine, Jena University Hospital, Am Klinikum 1, 07747 Jena, Germany; 4 Department of Nuclear Medicine, University of Leipzig Medical Centre, Liebigstrasse 18, 04103 Leipzig, Germany; 5 Friedrich Schiller University, Kastanienstrasse 1, 07747 Jena, Germany; 6 Jena Center for Soft Matter, Friedrich Schiller University, Philosophenweg 7, 07743 Jena, Germany

**Keywords:** Gallium-68, Positron emission tomography, Liver diagnostics, TARE, Liver function

## Abstract

**Background:**

The aim of this study was to investigate the preclinical biodistribution and molecular pathway of [^68^Ga]Ga-BP-IDA and to evaluate its clinical suitability for quantitative monitoring of liver function during transarterial radioembolization (TARE) therapy of a hepatocellular carcinoma (HCC).

**Results:**

[^68^Ga]Ga-BP-IDA undergoes hepatobiliary clearance, with uptake into hepatocytes via OATP1B1 and OATP1B3. [^68^Ga]Ga-BP-IDA exhibits demetallation in vivo but is nevertheless suitable for clinical application due to its rapid uptake into functional liver tissue. In a clinical case [^68^Ga]Ga-BP-IDA PET/CT allowed for differentiation of functional liver mass from cancerous tissue and enabled monitoring the effect on liver and tumor volume as well as on residual liver function after TARE therapy. Following TARE treatment, a reduction of the hepatic uptake rate was observed in both non-cancerous liver lobes, but was more pronounced in the right lobe, indicating a correlation to the higher non-targeted radiation dose from the TARE treatment in this lobe. [^68^Ga]Ga-BP-IDA PET/CT thus revealed additional information on liver function impairment which was not represented by CT-based volumetry alone.

**Conclusion:**

[^68^Ga]Ga-BP-IDA PET/CT is a suitable tool for planning and monitoring TARE therapy of primary liver tumors and may complement the limits of volumetry-based methods with functional information about the liver.

**Supplementary Information:**

The online version contains supplementary material available at 10.1186/s13550-025-01327-2.

## Background

Non-invasive liver function determination is a crucial factor for the success of liver cancer treatment. Information on the pre- and post-therapy liver function distribution and function restoration processes are of particular importance during procedures featuring targeted treatment of a specific liver region. Transarterial radioemblization (TARE) is an established locoregional treatment option for primary and secondary liver malignancies. During a TARE procedure, microspheres containing a therapeutic radionuclide are injected into arteries supplying the liver regions containing the tumor(s) on a lobar or segmental level. Because of the primarily arterial tumoral blood supply, microspheres accumulate predominantly in the hyperperfused tumor region and irradiate the cancer cells, but damage to non-tumoral liver tissue in the TARE target area cannot be avoided completely. So far, TARE treatment response is monitored by visualising tumor shrinkage by computed tomography (CT) or magnetical resonance imaging (MRI). In some cases it may depict shrinkage of the treated liver lobe and hypertrophy of the contralateral lobe, but the impact of the procedure on the liver function beyond morphological changes cannot be determined. Novel hepatocyte-specific tracers for positron emission tomography (PET) may be suitable companion diagnostics for TARE, allowing to derive information on the distribution of liver function.

So far, the only radiopharmaceuticals applicable for liver function determination are scintigraphic tracers, the most common being [^99m^Tc]Tc-mebrofenin. A protocol for liver function evaluation by means of hepatobiliary scintigraphy (HBS) was established in the 1990 s and consecutive studies proved its feasibility, e.g. for estimation of functional liver reserve prior to planned hepatectomy [1–9]. However, HBS is not used frequently, likely due to the limited spatial resolution and quantifiability of the technique, which is especially hampered by planar (two-dimensional) dynamic imaging. In contrast, PET is a particularly suitable technique for the calculation of imaging-based functional parameters due to its inherent quantifiability and high spatio-temporal resolution. The development of PET radiopharmaceuticals of high hepatobiliary specificity may provide a complementary diagnostic tool, enabling precise liver function monitoring during liver treatments. PET tracers based on gallium-68 feature wide-spread availability due to in-house radionuclide production from generators or cyclotrons and fast and efficient radiolabeling. Some promising classes of substrates suitable for hepatocyte targeting are bile acid analogues [10, 11] and liver-specific dyes like indocyanine green [12] and bromosulfophthaleine [13]. In the 1980 s, a brominated phenolphthaleine derivative, 4,5,6,7-tetrabromo-o-cresolphthalein-3′-methyliminodiacetic acid (BP-IDA), was described as a liver-specific chelator for gallium-68 and the tracer was evaluated in rats and in two healthy volunteers [14]. However, following its discovery [^68^Ga]Ga-BP-IDA has not been used for nearly four decades, likely due to the limited availability of the PET technology and thus the little significance of gallium-68 at the time. Recently, we have presented the re-establishment of [^68^Ga]Ga-BP-IDA production according to Good Manufacturing Practice (GMP) standards, along with a case report verifying the tracers‘ specificity towards hepatocytes [15].

To further investigate the potential of [^68^Ga]Ga-BP-IDA, we have undertaken more extensive preclinical experiments intended to reveal the molecular mode-of-action and in-vivo stability of the tracer. Additionally, [^68^Ga]Ga-BP-IDA PET/CT was employed for the monitoring of liver function in a patient with hepatocellular carcinoma (HCC) who underwent two consecutive TARE procedures.

## Methods

### Radiotracer synthesis and preclinical studies

[^68^Ga]Ga-BP-IDA was prepared according to GMP guidelines as previously reported [14, 15]. [^99m^Tc]Tc-mebrofenin was prepared according to the manufacturer’s instruction, using a commercial kit (Medi Radiopharma, Érd, Hungary) and [^99m^Tc]Tc-pertechnetate solution from a commmercial ^99^Mo/^99m^Tc-generator (Curium Netherlands B.V., Petten, The Netherlands). Detailed protocols on tracer production and analysis, the preclinical studies, determination of stability and lipophilicity, hepatocyte transporter cloning and transporter binding studies are provided in the supplementary information [16]. 

## Clinical application

Dynamic [^68^Ga]Ga-BP-IDA PET/CT was performed in a patient who was diagnosed with hepatocellular carcinoma (HCC; single lesion, cT2 cN0 cM0, G1, no liver cirrhosis) before (week 4) and after (week 85) two transarterial radioembolization (TARE) procedures. TARE 1 was performed as a sequential-bilobar treatment in week 4 (left lobe) and week 8 (right lobe). A second TARE was performed in week 32 (microsphere injection from two catheter positions: segment IV artery and right hepatic artery, complete tumor coverage, segments II/III spared). TARE treatments were done with ^166^Ho-loaded poly-L-lactic acid (PLLA) microspheres (QuiremSpheres^®^, Terumo, Leuven, Belgium). Energy doses delivered to the tumor and the non-targeted liver lobes were determined via pretherapeutic evaluation with SPECT/CT using a scout dose according to the manufacturer’s specifications.

The mean administered activity of [^68^Ga]Ga-BP-IDA was 136 ± 2.8 MBq (range, 134–138 MBq). The mean and standard deviation of the administered mass of BP-IDA precursor in the injection solutions was 10.3 ± 3.2 µg (range, 8.0–12.5 µg). There were no adverse or clinically detectable pharmacologic effects in any of the administrations to the subject.

Detailed protocols on PET/CT acquisition and image analysis are given in the supplementary information.

## Results

### Stability and lipophilicity

[^68^Ga]Ga-BP-IDA exhibited limited stability in PBS (pH = 7.4) and human serum (fig. S1 and fig. S2, supplementary information). When diluted with PBS, the RCP had decreased to 57.0% (ESI) after one hour due to significant demetallation. Comparable demetallation occured during incubation in human serum (fig. S1 and fig. S2). When diluting the tracer with saline (0.9%, pH = 6.1), demetallation occured much slower with RCP = 94.6% after 55 min (fig. S3). Correspondingly, metabolite studies in ostrich embryos revealed radiotracer demetallation in all investigated tissues (fig. S4). Notable demetallation was visible in blood serum and kidney homogenate. Liver tissue extracts showed that in hepatocytes about 75% of the tracer is still intact at 60 min p.i., with notable demetallation and an unknown metabolite (7.5 min) being detectable. A similar degree of decomposition was found in the gastric content. Analysis from intestines revealed significant demetallation, however, it was found that the percentage of ionic Ga-68 in the intestinal fluid sample increased over time, implying that demetallation continued in the sample post-mortem (fig. S4). In a metabolite study of [^99m^Tc]Tc-mebrofenin in ostrich embryos, in all samples (liver and kidney homogenate, intestinal and gastric fluid) no radiosignal at all was detected on HPLC (background only), while in radio TLC analysis for both, system A and system B, almost all radiotracer activity was found at the bottom of the TLC plate (R_f_ = 0) (fig. S5).

The logP of [^68^Ga]Ga-BP-IDA as determined via shake-flask method was 1.9 ± 0.2. The logP of [^99m^Tc]Tc-mebrofenin as determined via the same method was − 0.3 ± 0.1.

## Hepatocyte transporter binding studies

**Fig. 1:**
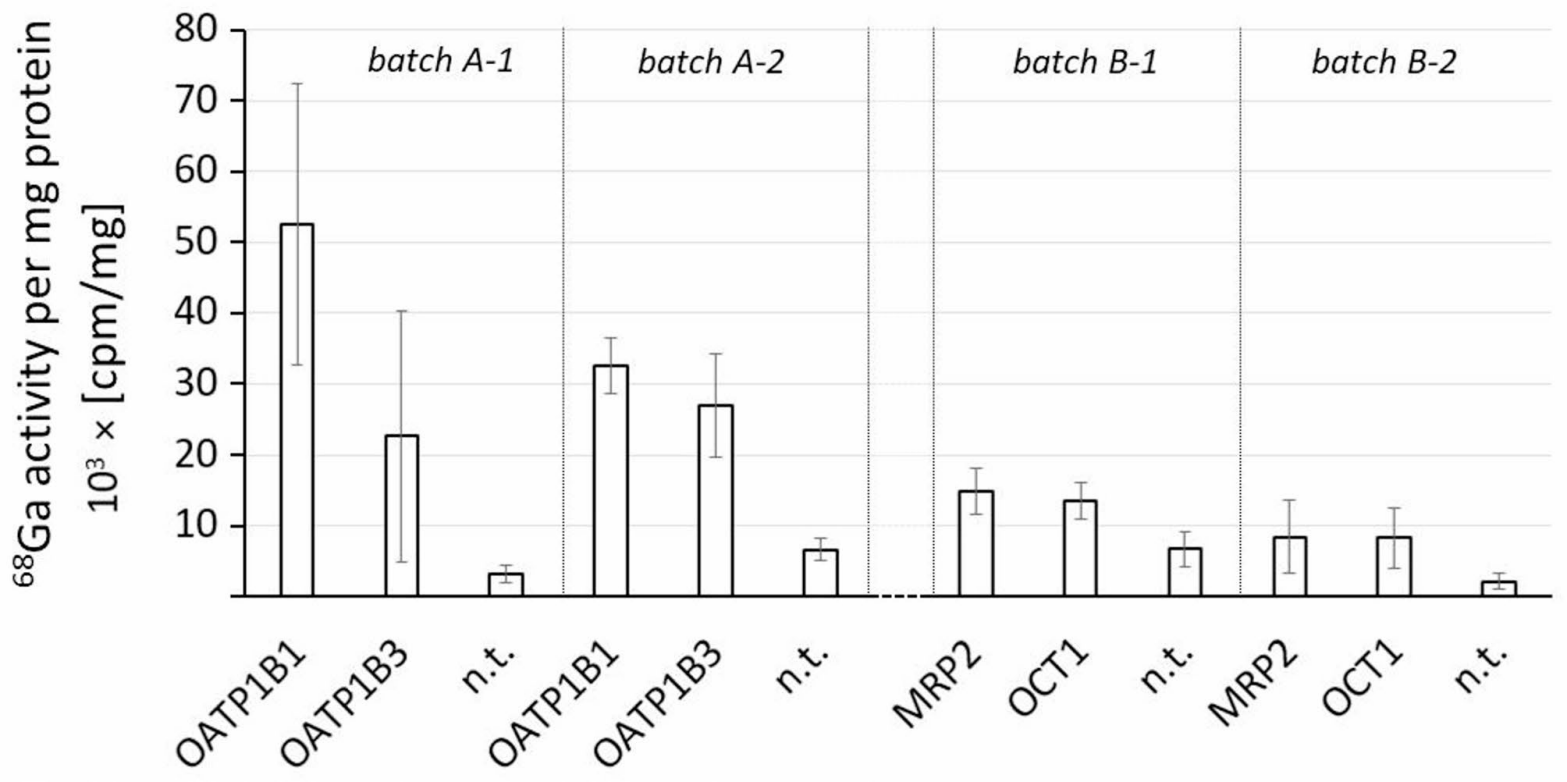
Mean bar plot of the [^68^Ga]Ga-BP-IDA activity in 0.1 × 10^6^ transfected HEK293t cells expressing four different transporters compared to the activity in the respective non transfected (n.t.) HEK293t cells as controls. Activity is given in counts per minute (cpm) per mg protein. Columns indicate the mean value, the standard deviation is indicated by error bars (*n* = 6). Each experiment was done with two different batches of tracer and two independent batches of transfected cells

For comparison with [^99m^Tc]Tc-mebrofenin, a known substrate for OATP1B1, OATP1B3 and MRP2, these transporters were chosen for binding studies with [^68^Ga]Ga-BP-IDA [17, 18]. [^68^Ga]Ga-BP-IDA exhibited statisticially significantly higher uptake in HEK293t cells expressing transporters OATP1B1, OATP1B3, MRP2 and OCT1, in all tested batches (Fig. 1). Uptake in OATP1B1 and OATP1B3 cells was higher than in MRP2 and OCT1 transfected cells, however, the uptake in MRP2 and OCT1 transfected cells was still significantly higher than in the respective controls in each batch, according to t test.

**Fig. 2:**
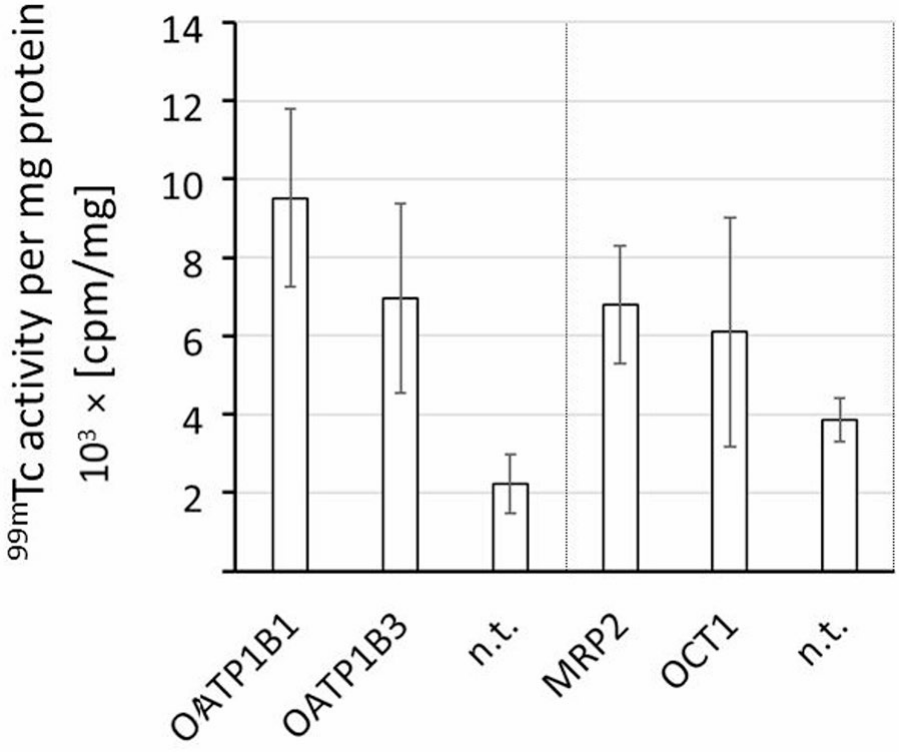
Mean bar plot of the [^99m^Tc]Tc-mebrofenin activity in 0.1 × 10^6^ transfected HEK293t cells expressing four different transporters compared to the activity in the respective non transfected (n.t.) HEK293t cells as controls. Activity is given in counts per minute (cpm) per mg protein. Columns indicate the mean value, the standard deviation is indicated by error bars (*n* = 6)

[^99m^Tc]Tc-mebrofenin exhibited statisticially significantly higher uptake in HEK293t cells expressing transporters OATP1B1, OATP1B3 and MRP2, and not quite statisticially significant uptake in HEK293t cells expressing OCT1, according to t test (Fig. 2). The experiment was performed once as a reference standard to verify successful transporter transfection and for comparison of the transporter binding profile of [^68^Ga]Ga-BP-IDA with the the already reported transporter binding profile of [^99m^Tc]Tc-mebrofenin [17].

### Preclinical biodistribution and pharmacokinetics

**Fig. 3:**
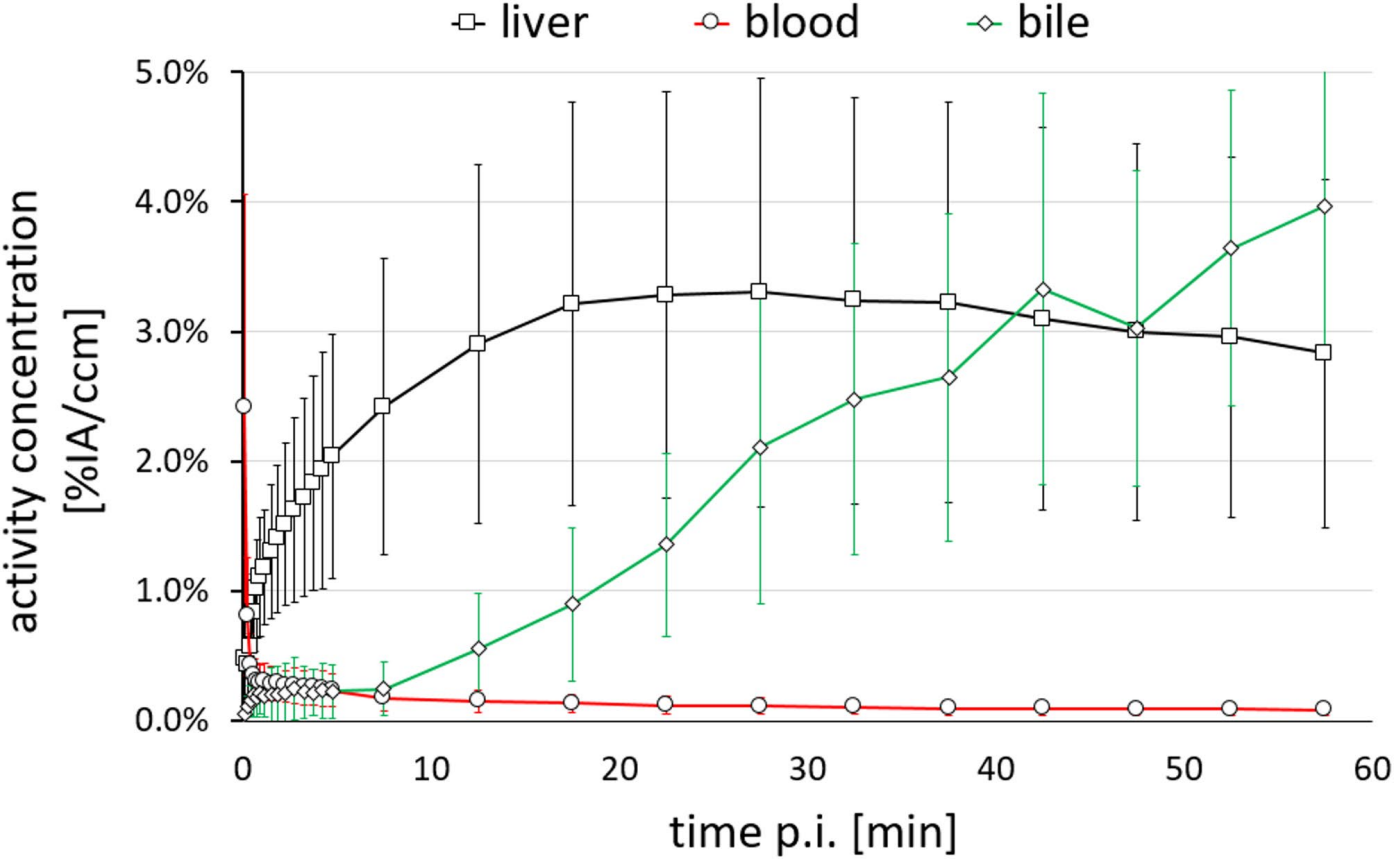
Time activity curve of [^68^Ga]Ga-BP-IDA activity concentration (%IA/ccm) in the liver, at the bile duct and in the blood of ostrich embryos post injection (p.i.); given as the mean (*n* = 7). The standard deviation is indicated by error bars

[^68^Ga]Ga-BP-IDA was cleared rapidly from the blood and exhibits a fast uptake into the liver of ostrich embryos, reaching a maximum activity concentration between 15 and 30 min p.i. after which a slow decrease can be observed due to biliary excretion (Fig. 3). Activity in the bile channel could be detected from about 10 min p.i. onwards, exhibiting a continuous increase until 60 min p.i.

**Fig. 4:**
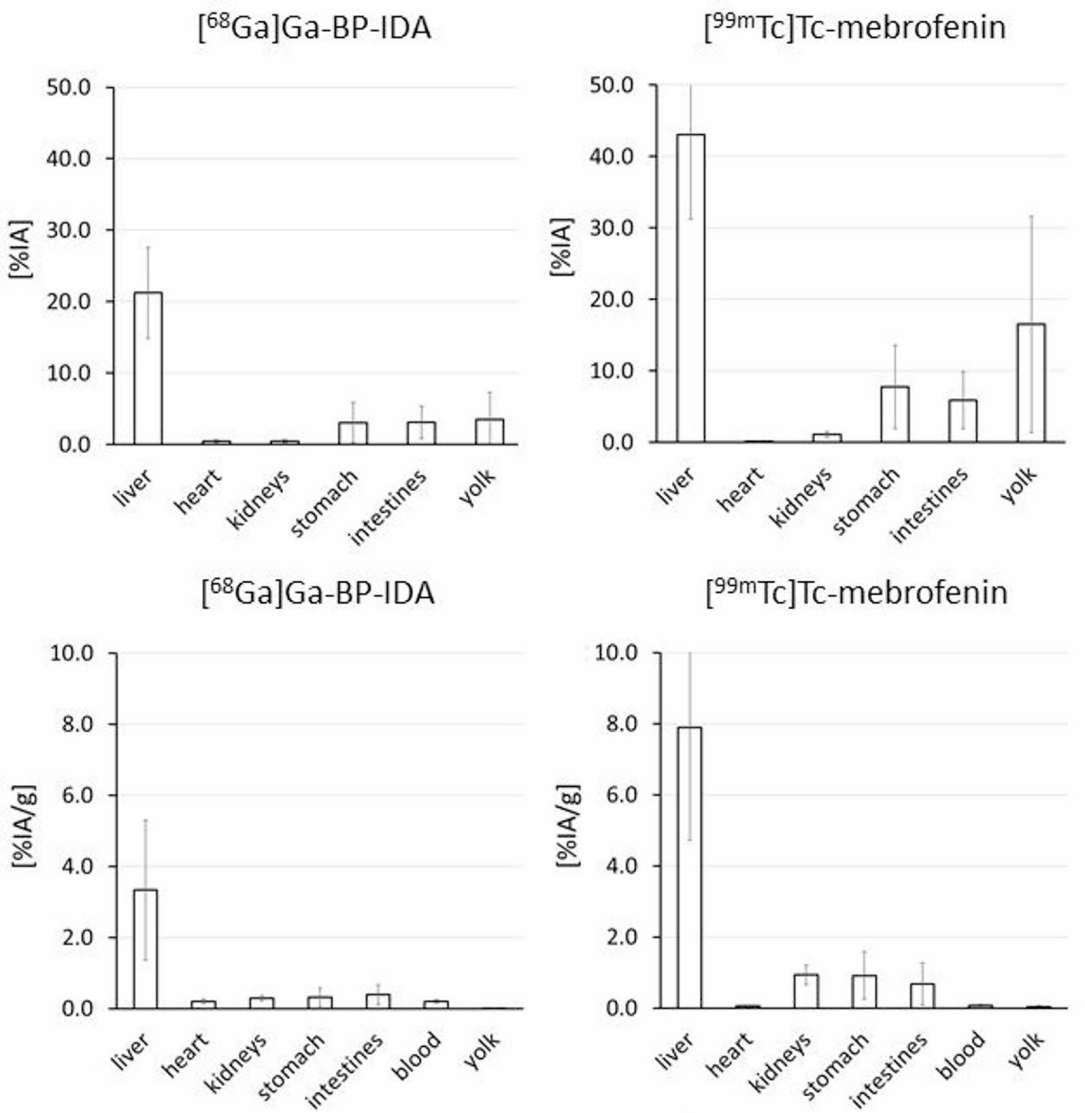
Biodistribution of [^68^Ga]Ga-BP-IDA (*n* = 7) and [^99m^Tc]Tc-mebrofenin (*n* = 5) in ostrich embryos at 60 min p.i., given as mean values of uptake (%IA, top) and of %IA/g (bottom). The standard deviation is indicated by error bars

Biodistribution studies confirmed high liver uptake of [^68^Ga]Ga-BP-IDA at 60 min p.i. (Fig. 4). Distinctly less activity concentration was found in the kidneys, stomach and intestines, even less was found in the blood compartment and almost no activity was found in the yolk. Ex vivo activity at 60 min p.i. was 21.2 ± 6.3%IA in the liver and 3.1 ± 2.3%IA in the intestines. For reference, the biodistribution of established HBS tracer [^99m^Tc]Tc-mebrofenin was studied in the same model. [^99m^Tc]Tc-mebrofenin exhibited about twice the uptake in the liver (43.1 ± 11.8%IA) and intestines (5.9 ± 4.0%IA) compared to [^68^Ga]Ga-BP-IDA (Fig. 4).

## Hepatic uptake rate of [^68^Ga]Ga-BP-IDA before and after TARE treatments

**Fig. 5:**
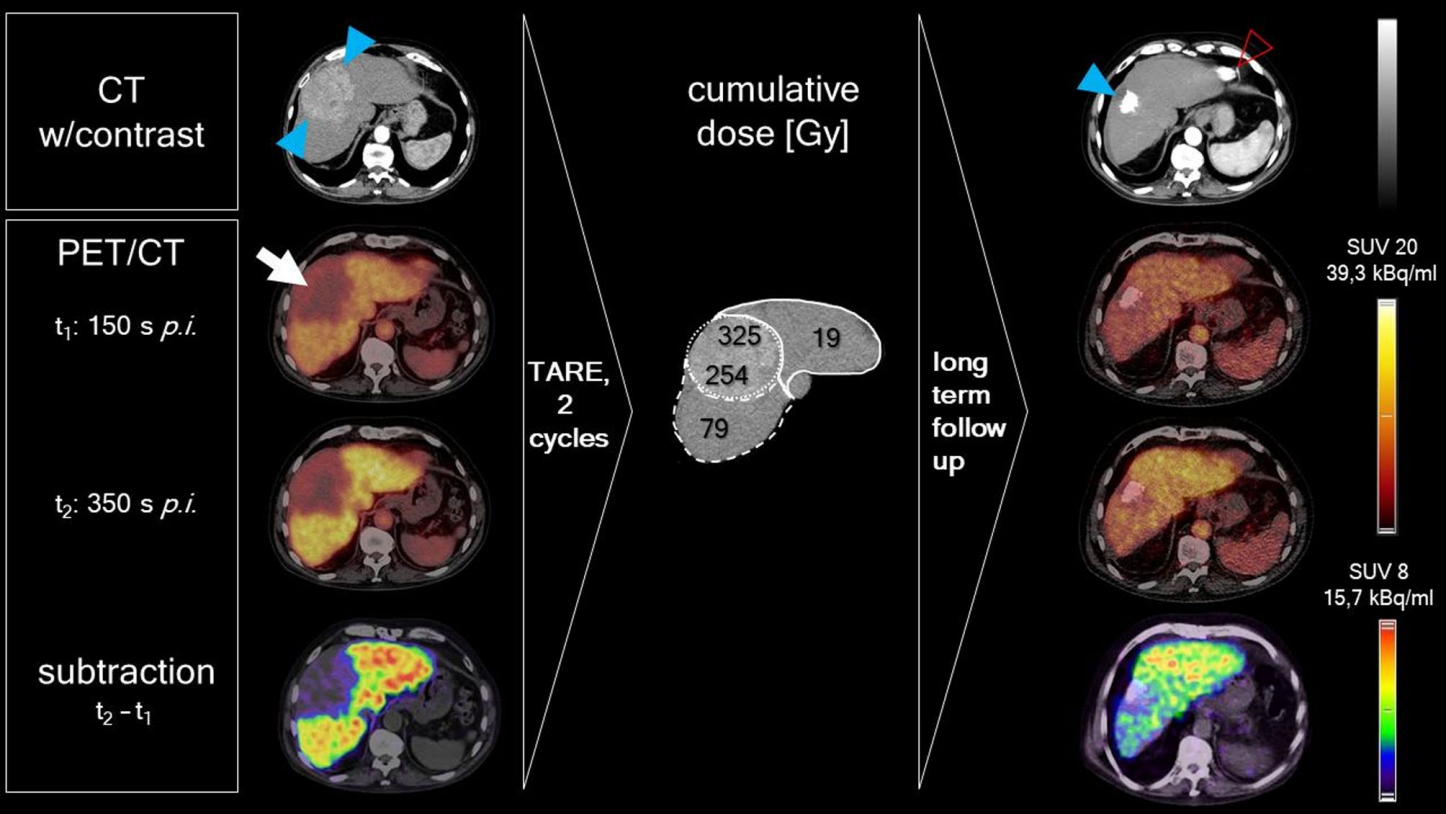
The solitary HCC of the 82-year-old patient involved both liver lobes. Contrast-enhanced CT (upper row) and [^68^Ga]Ga-BP-IDA-PET/CT (second row 150 s *p.i.*; third row 350 s *p.i.*) were performed before and after two TARE treatments. The hypervascular HCC (blue arrowheads) is seen as an area of low tracer uptake (white arrow). Subtraction of the early (150 s *p.i.*) PET dataset from the late (350 s *p.i.*) PET dataset gave a subtraction image visually highlighting the increase in activity concentration (bottom row). TARE treatment of the left and right liver achieved complete remission of the tumor with hyperdense areas corresponding to previous microsphere accumulations (blue arrowhead). The cumulative energy doses in [Gy] in the tumor area and in the non-targeted lobes are denoted by black numbers in the middle. On the CT after TARE, the heart is visible (red arrowhead)

In the reported patient, [^68^Ga]Ga-BP-IDA accumulated within the non-tumorous liver tissue, while the hepatocellular carcinoma (HCC) appeared as area of low, but still definite activity (Fig. 5). The tumor-related storage defect of [^68^Ga]Ga-BP-IDA, i.e. the area of impaired liver function was slightly larger than the hypervascular HCC depicted on contrast-enhanced CT (Fig. 5, upper row), which may indicate a margin of tumor infiltration not yet visible on CT.

The two consecutive TARE therapies resulted in high cumulative doses delivered to the tumor (right and left lobar parts, 325 and 254 Gy, respectively, Fig. 5). Tumor-free areas of the right and left liver lobes received cumulative radiation doses of 79 and 19 Gy, respectively. The tumor volume decreased from 580 ccm to 32 ccm (94% reduction), the residuum probably mostly consisting of scar tissue. Areas of high microsphere density are commonly seen as hyperdensities on follow-up CT after ^166^Ho-based TARE due to the high x-ray attenuation of residual holmium. In accordance with tumor volume reduction, the storage defect visible on the [^68^Ga]Ga-BP-IDA PET/CT images (both at 150 s and 350 s p.i.) in the area of tumor decreased in size after TARE treatment. Initially, both liver lobes show similar and rather homogeneous distribution of the tracer (Fig. 5). After TARE treatments the activity concentration is reduced in both lobes, with the activity concentration in the right lobe being lower than in the left lobe. This difference can be seen on both PET/CT images, at 150 s and 350 s p.i., and also on the subtraction image visualizing the activity concentration increase between the two time points (Fig. 5).

No additional oncologic therapies were applied. The liver function parameters of the patient remained stable, with a Child-Pugh score of A5 and an ALBI grade 1 (−3.14 pre-TARE, −2.75 at follow-up).

**Fig. 6:**
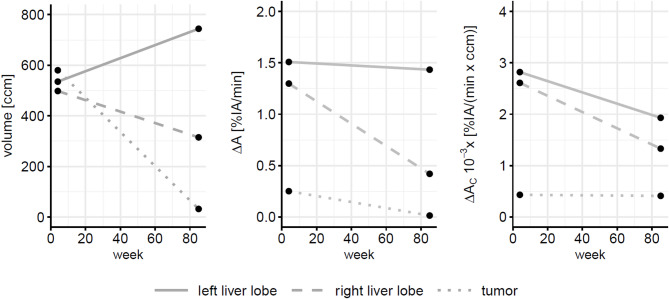
Changes of volumes (left diagram), of activity increase between 150–350 s *p.i.* (ΔA, middle diagram), and of the increase in activity concentration between 150–350 s *p.i.*, (ΔA_c,_ right diagram), in the tumor and in the tumor-free areas of the liver at weeks 4 and 85

While the tumor size was reduced after TARE, the volume of the tumor-free left liver lobe (segments II/III) increased from 535 ccm (week 4) to 744 ccm in week 85, while the volume of the tumor-free right liver lobe decreased from 498 ccm to 315 ccm (Fig. 6, left diagram). The percentage of injected activity taken up between 150 s and 350 s p.i. by the respective region (ΔA) remains constant in the left lobe, but is markedly reduced in the right lobe and also in the tumor after TARE (Fig. 6, middle). The difference in activity concentration (ΔA_C_) is reduced in both liver lobes after TARE, with the right lobe exhibiting slightly more reduction (Fig. 6, righthand side). Compared to the tumor-free liver tissue, the tumor shows very low ΔA_c_ at both time points, in accordance to the area of reduced intensity visible on the PET/CT and on the subtraction images.

## Discussion

[^68^Ga]Ga-BP-IDA is a lipophilic substrate derived from tetrabrominated phenolphthaleine, which is chemically related to bromophenol blue, a dye that also exhibits hepatobiliary clearance in correlation to the functional state of hepatocytes [19, 20]. The logP of 1.9 ± 0.2 determined in this study is comparable to the result reported by Schuhmacher et al. (logP = 2.1 [14]). A range of hepatocyte-targeting tracers and their respective logP values have been reported, particularly for [^99m^Tc]Tc-labeled IDA substrates. As a general rule, a higher logP value and thus a higher lipophilicity is expected to increase hepatic uptake and reduce renal excretion, while at the same time a high lipophilicity and bulky substituents like isopropyl can result in slow biliary excretion from hepatocytes and prolonged liver retention [21, 22]. However, studies correlating chemical structure and biodistribution repeatedly showed that there is no simple linear relationship between lipophilicity and a suitable hepatobiliary profile, because additional parameters like steric effects, compound charge, and position of aromatic substituents may strongly influence hepatobiliary pharmacokinetics [21, 22]. [^99m^Tc]Tc-mebrofenin, with one of the highest liver uptakes among known radiotracers, exhibited a log P of −0.3 as determined in our study, and a logP of −0.58 to −0.70 as determined by a comparable extraction method by Brboric et. al. [21], indicating much lower lipophilicity than [^68^Ga]Ga-BP-IDA. Nevertheless, our biodistribution studies show that [^99m^Tc]Tc-mebrofenin exhibits a superior hepatobiliary profile compared to [^68^Ga]Ga-BP-IDA, in particular a higher liver uptake (vide infra). This underlines the limited predictability of biokinetics based solely on logP.

In biodistribution and kinetic PET studies in ostrich embryos [^68^Ga]Ga-BP-IDA exhibits a specific hepatobiliary excretion profile with high liver uptake and only small amounts undergoing renal excretion. The presence of notable amounts of radiotracer in the stomach was also observed for [^99m^Tc]Tc-mebrofenin and other hepatocyte-targeting radiotracers when using this preclinical model [23]. We hypothesize that this finding is due to the stomach partaking in a gastrointestinal reflux of bile containing the radiotracer. Due to the lack of a gall bladder, the radioactivity excreted from the liver cells into the bile channel flows into the small intestine directly underneath the gastric connection to the intestine.

Ex-vivo quantification indicates that in total about 25% of the injected [^68^Ga]Ga-BP-IDA activity are excreted via the hepatobiliary route after one hour, with the most activity still being retained in the liver. Correspondingly, the dynamic PET studies indicate slow biliary excretion and significant retention of tracer activity in the liver. In contrast, Schuhmacher et al. reported a much faster clearance in rats, with a maximum of 60%IA in the liver at 6 min p.i. and only 11%IA at 60 min p.i [14]. These differences may be explained by different metabolic rates between rats and ostrich embryos. As previously shown in a dynamic PET in a patient, the excretion rate of [^68^Ga]Ga-BP-IDA from human liver is also slower compared to rats, with about 42%IA at 18 min p.i. and about 37%IA still in the liver at 60 min p.i [15].

In our study, the established HBS agent [^99m^Tc]Tc-mebrofenin showed about twice as high uptake in liver and intestines of ostrich embryos compared to [^68^Ga]Ga-BP-IDA. Additional to exhibiting exceptionally high liver uptake, [^99m^Tc]Tc-mebrofenin also exhibits the fastest biliary excretion rate among the group of [^99m^Tc]Tc-IDA compounds [24]. In our ostrich embryo study for both [^68^Ga]Ga-BP-IDA and [^99m^Tc]Tc-mebrofenin the ratio of activity in the intestines in relation to the activity in the liver was similar (14–15%) at 60 min p.i., indicating comparably slow biliary clearance of both tracers in the ostrich embryo model. However, this preclinical model seems to be of limited suitability to adequately predict biliary excretion rates of radiotracers in the human situation. For comparison, in a normal [^99m^Tc]Tc-mebrofenin scan in human the liver parenchyma contains less than 25% of the maximum counts at 60 min p.i., with most of the activity being found in the biliary and intestinal tract, indicating faster elimination than [^68^Ga]Ga-BP-IDA, which showed higher activity retention in the liver at 60 min p.i. in a reported clinical case (37%IA [15, 24]). Whether [^68^Ga]Ga-BP-IDA consistently exhibits slower biliary excretion compared to [^99m^Tc]Tc-mebrofenin in human would need to be verified by clinical data in a larger cohort.

Incubation studies in HEK cells transfected with hepatocyte transporters were validated by using [^99m^Tc]Tc-mebrofenin as a reference standard. The binding profile revealed specificity towards OATP1B1, OATP1B3 and MRP2, which is in accordance to transporter study results gained by other groups [17, 18, 25]. Our transporter binding studies show that [^68^Ga]Ga-BP-IDA too is a substrate for OATP1B1 and OATP1B3 as well as for MRP2 and OCT1. The specificity towards OATP1B1, OATP1B3 and MRP2 make it comparable to [^99m^Tc]Tc-mebrofenin [17] and substantiate the previous findings by Schuhmacher et al., who reported that bilirubin and bromosulphthaleine inhibit [^68^Ga]Ga-BP-IDA uptake [14]. The binding to OCT1 is surprising given that a negative net charge was reported for [^68^Ga]Ga-BP-IDA at pH 7.2 [14]. However, recently some atypical substrates, partially exhibiting neutral and anionic charges, where reported to exhibit binding to OCT1 as well, which indicates that despite its name, OCT1 is not exclusive for cationic substrates [26].

Due to demetallation, [^68^Ga]Ga-BP-IDA has a limited shelf-life when diluted with PBS. Thus, the tracer should only be diluted with saline and used as soon as possible after production. The limited stability is likely related to the chelator structure: Schuhmacher et al. postulated radiometal coordination via two oxygen donors and a nitrogen donor from the iminodiacetic acid groups added by a neighbouring phenol oxygen donor. This results in a tetracoordination from the ligand, added by two water molecules completing the hexavalent coordination sphere, with an overall negative net charge resulting from hydrolytic cleavage of the lactone group [14]. It is generally presumed that Ga(III) complexes are particularly stable when chelators enable hexacoordination and that a reduction in number of coordinating sites reduces the compounds‘ vivo stability.

Metabolite analysis from ostrich embryo tissues confirmed demetallation of [^68^Ga]Ga-BP-IDA in vivo, which was particularly notable in blood samples and kidney extracts (fig. S4). Liver, gastric fluid and intestinal fluid content revealed an unknown radiometabolite at 7.5 min and also some demetallation, as has been described before by Schuhmacher et al. [14]. Surprisingly, we found that the degree of demetallation in intestinal fluid samples increased over time, as shown by repeatedly injecting the same probe into the radio HPLC. This implies that demetallation may continue post-mortem. Despite the limited stability, a substantial percentage of the injected activity of [^68^Ga]Ga-BP-IDA is taken up by the liver tissue, both in the preclinical studies and in the clinical case reported herein.

In our metabolite study of [^99m^Tc]Tc-mebrofenin in ostrich embryos, no radiotracer signal was detected on radio HPLC, indicating strong binding of [^99m^Tc]Tc-mebrofenin to colloidal or bulky structures like proteins which prohibits migration over the HPLC column. Likewise, radio TLC consistently showed most radiotracer activity at the bottom of the TLC plate (R_f_ = 0) when using system B wherein [^99m^Tc]Tc-mebrofenin migrates to the top (fig. S5). This too points to potentially strong binding of the tracer to a bulky substrate prohibiting migration on the TLC plate. While hypothetically [^99m^Tc]Tc-colloid formed by tracer decomposition, would show the same retention on TLC, [^99m^Tc]Tc-colloid would be stored in liver macrophages and the spleen and not exhibit biliary excretion, thus we consider it more like that the [^99m^Tc]Tc-mebrofenin is bound to a biomacromolecule. [^99m^Tc]Tc-mebrofenin is known to bind strongly to human serum albumin in the blood [21], however it remains unclear which compounds in the liver, bile and gastrointestinal tract could be responsible for [^99m^Tc]Tc-mebrofenin binding in our study. While it has been reported that [^99m^Tc]Tc-mebrofenin is being excreted from the liver unmetabolised, no original data on compound identification and composition analysis was provided [27].

In the case reported here, by qualitative image analysis alone, [^68^Ga]Ga-BP-IDA PET/CT was suitable to depict the functional regions within the liver, helping to delineate the non-functional, cancer-affected area and thus allowing for monitoring of the effect of TARE treatments. For quantitative evaluation we implemented a PET-based protocol in analogy to the Ekman formula established for HBS [1], wherein the increase of activity concentration in the liver or in a respective liver segment between two time-points (t_1_ = 150 s, t_2_ = 350 s) is determined as a measure of the hepatic uptake rate. The quantitative PET derived parameter ΔA_c_ (%IA/ccm/min) is suitable to depict the hepatic uptake rate of the tracer irrelevant of the volume of the investigated lobe or segment and irrelevant of total injected activity. Previous reports verified a correlation between the hepatic uptake rate of the established HBS agent [^99m^Tc]Tc-mebrofenin and biochemical factors used to evaluate liver function, like ALT, AST and bilirubin [28]. Since [^68^Ga]Ga-BP-IDA shows similar hepatocyte transporter binding like [^99m^Tc]Tc-mebrofenin we assume that similarly a higher [^68^Ga]Ga-BP-IDA ΔA_c_ value correlates with a higher liver function.

In the reported case, CT volumetry showed hypertrophy of the tumor-free areas of the left liver lobe, which received only a low non-targeted radiation dose during the first TARE, as a compensatory reaction to the shrinkage of the right lobe. The increase in volume was able to compensate the decrease of excretory function per ccm liver tissue (ΔA_c_), so that the total activity of metabolized radiotracer per minute (ΔA) remained unchanged before and after TARE (Fig. 6, middle diagram). From these results, it is also evident that liver hypertrophy does not always lead to an increase of liver function. Correspondingly, the tumor-free regions of the right lobe which received a higher cumulative non-targeted radiation dose from TARE exhibited a relative decrease in excretory function that exceeded their relative decrease in volume, indicating a more profound tissue damage. Therefore, in the case presented, CT volumetry overestimated the positive functional effect of hypertrophy but underestimated the negative functional effect of hypotrophy.

In general, volume-based information are hard to interpret without correlating the segmental volume to functional parameters. That liver volume and liver function do not necessarily correlate is a well-known problem in procedures involving portal vein ligation for staged hepatectomy (ALPPS), wherein solely volume-guided determination of functional liver reserve can be insufficiently precise [9, 29]. Our results highlight the importance of additional functional parameters to be implemented in liver function protocols used for planning TARE treatment but also partial hepatectomies.

For TARE treatments, a positive relationship has been established between delivered dose to the tumor and treatment response, resulting in a tendency of dose escalation [30–32]. However, the relationship between the dose applied to the healthy (non-tumoral) tissue and TARE toxicity is not yet completely clear, regarding cumulative doses in particular [33, 34]. Patients with liver cirrhosis already have an increased mortality correlating with liver function impairment, and for TARE planning, finding the balance between tumor treatment and risk of liver failure is of utmost importance. Established liver functional scores like Child-Pugh and Albumin-Bilirubin (ALBI) scores, reflecting global liver function are helpful, but not yet sufficient when considering patients for locoregional treatment. Our patient, who did not suffer from liver cirrhosis, laboratory liver function parametes remained stable despite the obvious changes seen on imaging. Liver function PET/CT may close this gap because it allows loco-regional liver function measurements, complementing the advances brought by personalized dosimetry techniques and sophisticated, more accurate microsphere injection system allowing exact sub-segmental treatments [35, 36].

[^68^Ga]Ga-BP-IDA is a feasible hepatocyte-targeting tracer for evaluating changes in the hepatic blood clearance efficiency. Significant demetallation of the tracer may occur in cases of prolonged blood circulation time due to limited hepatocyte extraction, e.g. in cases of high levels of bilirubin, which is known to affect hepatic clearance due to competitive transporter blocking [14]. Definite advantages of this hepatobiliary PET tracer are, compared with scintigraphy and SPECT/CT, much higher spatial and particularly temporal resolutions, with the possibility of voxel-based evaluation of molecular liver functions. The approach may improve patient selection for, and planning and surveillance of segmental or sub-segmental locoregional hepatic tumor therapies.

## Conclusion

[^68^Ga]Ga-BP-IDA is a hepatocyte-targeting tracer suitable for monitoring changes in the hepatic blood clearance efficiency and thus differentiating functional liver parenchyma from non-functional tissue. Dynamic [^68^Ga]Ga-BP-IDA PET/CT enables monitoring the effect of TARE treatments on the non-tumor tissue, thus adding a function-based dimension to established CT-based volumetry and potentially allowing for a more precise HCC treatment planning via TARE or partial hepatectomy in the future.

## Supplementary Information

Supplementary Material 1.

## Data Availability

More extensive data on methods and metabolite analysis are available as electronic supplementary material. Further datasets used and/or analysed during the current study are available from the corresponding author on reasonable request.
