# Supplementary material for: Hepatobiliary PET with [68Ga]Ga-BP-IDA – preclinical evaluation and its translational potential for liver function monitoring
Source: EJNMMI Res. 2025 Oct 2;15:128. doi: 10.1186/s13550-025-01327-2 (PMC12491138; doi:10.1186/s13550-025-01327-2)
Supplement: Supplementary file 1 — Supplementary Material 1. [file 13550_2025_1327_MOESM1_ESM.docx]

**Electronic supplementary information (ESI)**

Hepatobiliary PET with [^68^Ga]Ga-BP-IDA – preclinical evaluation and its translational potential for liver function monitoring

**Materials and Methods**

**Radiotracer synthesis**

The precursor BP-IDA was synthesized and subsequently labeled with Gallium-68 according to previously published procedures (*1,2*). In short, 400-1500 MBq of [^68^Ga]Ga-chloride solution (0.1 M, 5 mL) eluted from a Germanium-68/Gallium-68 generator (Eckert&Ziegler AG) were purified via cation exchange cartridge (PS‑H+, Macherey Nagel) using sodium chloride (5 M, 1 mL) for elution. The concentrated [^68^Ga]Ga-chloride solution and BP-IDA (30 µg) were combined in HEPES buffer solution (1.5 M, 3 mL) and labeled for 10 min at 100 °C. The tracer was purified using an SPE cartridge (C8 Light, Waters) and ethanol (1 mL, 60% v/v) for elution (*2*). The product was diluted with sterile saline (0.9%, 4 mL for preclinical injections, 15 mL for injections into humans). Quality control was performed as previously described (*2*). Radiochemical purity analysis involved radio HPLC (eurosphere 125x4 mm column (100-5 C18, Knauer); gradient: 0.0–2.5 min 97.0% A, 2.5–10.0 min 97.0% A → 0.0% A, 10.0–13.0 min 0.0% A, 13.0–13.05 min 0.0% A → 97.0% A, 13.05 – 16.0 min 97.0% A, A: water/trifluoroacetic acid (99.9%/0.1%), B: acetonitrile/trifluoroacetic acid (99.9%/0.1%)).

**Determination of in-vitro stability and lipophilicity (logP)**

For in-vitro stability studies, 50 µL of a solution of [^68^Ga]Ga-BP-IDA (10 MBq) in ethanol (1 mL, 60% v/v) were added to 500 µL of saline, PBS and human serum, respectively. Solutions in saline and PBS were kept at room temperature, and the human serum sample was incubated at 37°C. At dedicated time points samples were withdrawn for HPLC analysis. In case of the human serum samples an aliquot of the was centrifuged at 685 rcf (3500 rpm) for 5 min and the respective supernatant was injected for HPLC.

From a solution of [^68^Ga]Ga-BP-IDA (200 MBq) in ethanol (1 mL, 60% v/v), 60 µL (12 MBq) were diluted with 7940 µL saline, realizing an ethanol content < 0.5% (v/v). From this probe, 2.5 mL were mixed with n-octanol (2.5 mL) in a centrifugal tube and vigorously shaked using a vortex mixer (1500 rpm, 1 min). Then, the tube was centrifuged at 685 rcf (3500 rpm) for 5 min. Using a pipette, six aliquots were removed from the n-octanol layer (100 μL) and five aliquots from the water layer (400 µl), respectively, and each aliquot was measured in a gamma counter (ISOMED 2100, NUVIA Instruments, Dresden). The experiment was repeated three times.

**Hepatocyte transporter cloning**

The human coding sequences of *SLCO1B1* (NM_006446, Restriction Enzymes used: (RE): KpnI, NotI), *SLCO1B3* (NM_019844, RE: SacI, NotI), *ABCC2* (NM_000392, RE: HindIII, NotI), *SLC22A1* (NM_003057, RE: KpnI, NotI) coding for the hepatic transport proteins OATP1B1, OATP1B3, MRP2, and OCT1 were synthesized with individual restriction enzymes overhangs provided in brackets (GeneScript, USA). They were then cloned into a pcDNA3.1(+) Vector (LifeTechnologies, USA), that included an Ampicillin resistance under a prokaryotic promotor as well as an eucaryotic CMV promoter, an multiple cloning site, and N-terminal 6-His Tag for the inserted gene of interested. Vector transformed *E. coli* DH5α were cultivated for selection on LB-Agar Plates or LB liquid Medium (Carl Roth, Germany) under Ampicillin (Carl Roth, Germany) pressure (100 µg mL^-1^). The plasmids were isolated from liquid cultures using a commercial, column-based Kit (NucleoBond PC-100, Macherey Nagel, Germany). Restriction analysis with the above-mentioned restriction enzymes and sequencing confirmed the plasmids and correct insertion of the transgenes.

**Transporter binding studies**

HEK293t cells (DSMZ; ACC 635) were seeded in 12-well plates at a density of 0.1 × 10^6^ per well in 1 mL Dulbecco’s Modified Eagle’s medium (DMEM) supplemented with 10% fetal bovine serum (FBS) and 100 IU penicillin and 100 IU streptomycin and maintained in a tissue culture incubator at 37°C with 5% CO_2_. After 24 hours, the medium was replaced with Opti-MEM reduced serum medium (ThermoFisher Scientific). The cells were transfected using Lipofectamine™ 3000 following the manufacturer’s instructions (Invitrogen). Then, 6µg of transporter plasmids were mixed with 6µL P3000 reagent, 4.5µL of Lipofectamine 3000, and 300 µL Opti-MEM reduced serum medium. After incubating the mixture at room temperature for 15 minutes, 50 µL of the complex was added dropwise to each well, and cells were incubated for 48 hours.

For radiotracer uptake studies of [^68^Ga]Ga-BP-IDA and [^99m^Tc]Tc-mebrofenin, 10 kBq of the respective tracer in saline 0.9% (50 µL total) were added to each well containing transfected cells (n = 6) and to the wells containing non-transfected cells (n = 6), respectively. The plates were shaken gently for 10 min at 37°C using a shaker plate (160 rpm). Then, a cold solution of cyclosporine A in PBS (0.5 mM, 200 µL) was added to each well and the plates were put on ice. The well content was suspended and transfered into 24 separate Eppendorf tubes. The tubes were centrifuged at 4°C (0.3 rcf, 15 min). Then, from each Eppendorf tube the supernatant was carefully removed from the cell sediment. To each tube 200 µL of a cold solution of cyclosporine A in PBS (0.5 mM) were added. The sediment was resuspended with a pipette and then centrifuged at 4°C (0.3 rcf, 15 min). This washing procedure was repeated once. After removing the last supernatant fraction, 200µL of RIPA buffer (150 mmol/ NaCl, 1 mmol/L EDTA, 0.1% SDS, 1% Triton X-100, 500 mmol/L Tris-HCl, 0.5% deoxycholic acid) were added to the Eppendorf tubes, homogenized, and the probes were stored on ice. The activity in each Eppendorf tube containing the lysed cells was determined in counts per minute (cpm) using a gamma well counter (ISOMED 2100, NUVIA Instruments, Dresden, Germany). Subsequently, the protein concentration for each lysate was determined by BCA Protein Assay Macro Kit (Serva Gelelectrophoresis GmbH), using 10 µL non-diluted RIPA lysate and a dilution range of 2 mg/mL BSA stock solution as reference. The activity (in cpm) in each tube was divided by the respective protein content (in mg). Then, the mean activity per mg (in cpm/mg) was calculated for each group (n = 6). For each biological replicate, the experiment was repeated once. Statistical significance between the activity values in transfected and non-transfected cells was verified using the two-tailed standard t-test (α = 0.05).

**Preclinical studies**

Biodistribution studies of [^68^Ga]Ga-BP-IDA were performed on seven artificially incubated ostrich embryos on developmental day (DD) 37, according to a previously described procedure (*3*). Prior to imaging the embryos were immobilized using isoflurane (4%, 1 hour). After narcotization, the egg shell was removed at the site of the natural air cell using a rotating cutter. A vessel in the chorion-allantois-membrane was identified via candling and punctured using a 30G needle. Subsequently, the ostrich egg was placed on a standard clinical PET/CT scanner (Biograph mCT40, Siemens Healthineers, Erlangen, Germany) and the tracer was injected intravenously (11.0 ± 6.7 MBq). Dynamic PET was carried out in list mode, with acquisition starting simultaneously with the i.v. administration of the radiotracer and continuing for 60 mins. Image reconstruction was performed using the following time frames: 6 x 10s, 3 x 20s, 6 x 30s, and 11 x 300s. 60 min after imaging, the embryos were sacrificed *via* i.v. injection of pentobarbital (1.0 ml) and subsequent decapitation. For ex-vivo quantification of activity distribution, blood, egg yolk and organs were collected and weighed. The activity of the samples was measured using a gamma well counter or, in case of samples containing > 0.6 MBq, a dose calibrator (ISOMED 2100 or ISOMED 2010, NUVIA Instruments, Dresden, Germany).

PET image analysis was performed with PMOD software (v. 4.101, PMOD Technologies GmbH, Fällanden, Switzerland). For determination of kinetic tracer distribution, appropriate volumes-of-interest (VOI) were placed within the liver, over a prominent blood vessel and around the enterohepatic bile duct exit at the liver in place of the gall bladder, which is not developed in ostriches. Since the heart of the ostrich embryo is closely encased by the two prominently carved liver lobes, VOIs over the heart as a blood pool compartment are susceptible to activity flare from the surrounding liver tissue. Therefore, blood activity curves were derived from a VOI that was placed over a major blood vessel. Activity values in the VOIs were determined in kBq as average × volume.

For reference biodistribution studies, [^99m^Tc]Tc-mebrofenin (9.1 ± 1.3 MBq) was injected to five ostrich eggs using a similar protocol as described for [^68^Ga]Ga-BP-IDA, but foregoing the PET imaging. Ex-vivo quantification of organ activity was performed as described for [^68^Ga]Ga-BP-IDA.

For metabolite analysis of [^68^Ga]Ga-BP-IDA and [^99m^Tc]Tc-mebrofenin, the blood samples were centrifuged at 685 rcf (3500 rpm) for 5 min and the supernatant was used for radio HPLC analysis. Fluid content was collected from the intestines and from within the stomach and injected directly to HPLC. One liver and one kidney sample were homogenized in acetonitrile (ca. 3 mL per organ), respectively, using a small handheld electric mixer. The samples were centrifuged at 685 rcf (3500 rpm) for 5 min and the supernatant was injected to radio HPLC analysis. For [^99m^Tc]Tc-mebrofenin metabolite analysis, probes of intestinal fluid, stomach content, and liver and kidney homogenate were also used for radio TLC analysis, which was performed acccording to US Pharmacopeia (USP 29), using silicic acid-impregnated glass microfiber strips (ITLC-SA) and 10% sodium chloride solution (system A) for detection of [^99m^Tc]TcO_4_ impurity, and silica gel-impregnated glass microfiber (ITLC-SG) and acetonitrile/water (3/1, v/v, system B) for detection of [^99m^Tc]Tc colloidal impurities.

**Clinical application – PET/CT protocol and image analysis**

The patient was placed in a supine position in a Biograph mCT 40 PET scanner (Siemens Healthineers, Erlangen, Germany). A scout image of the upper abdomen for the required bed position was acquired. For attenuation correction, a low-dose CT (50 mAs, 120 kV, 3 mm slice thickness) was performed. Dynamic [^68^Ga]Ga-BP-IDA PET was carried out in list mode, with acquisition starting simultaneously with the i.v. administration of the radiotracer bolus and continuing for 30 mins.

Iterative image reconstruction containing point-spread function was performed with the TrueX HD software package (Siemens Healthineers, Erlangen, Germany). From the list mode data, two datasets were generated representing activity distribution at 150 s p.i. and 350 s p.i. (frames 120-180 s and 320-380 s, respectively). Image analysis was performed with Simplicit90Y software (Mirada Medical Ltd, Oxford, England) according to the following procedure: VOIs were drawn around the tumor and the non-tumorous left and right liver lobe in the CT frame. For the left lobe, the VOI only included segments II and III; since segment IV consisted exclusively of tumor. Then, for each of the two datasets at 150 s and 350 s p.i. the mean activity concentration A_c_ (in MBq/ccm) and volumes (in ccm) of the respective VOIs were determined. The activity concentration values at 150 s p.i. were subtracted from the activity concentration values at 350 s p.i, giving a value which represents the increase in activity concentration between 150 s and 350 s p.i in MBq/ccm*.* Then, this value was normalized to the total injected activity and given in %IA/ccm. Finally, this value was normalized to one minute, giving ∆A_c_ in %IA/ccm/min as a measure of hepatocyte function, in resemblance to a scintigraphy protocol established by Ekman et al. (*4*). PET subtraction images were constructed by subtracting the dataset at 150 s p.i. from the dataset at 350 s p.i. using HERMIA Gold Client HybridViewer (HERMES Medical Solutions, Stockholm).

**References**

**1.** Schuhmacher J, Matys R, Hauser H, Clorius JH, Maier-Borst W. A Ga-68-labeled tetrabromophthalein (Ga-68 BP-IDA) for positron imaging of hepatobiliary function: Concise communication. *Journal of Nuclear Medicine.* 1983;24:593-602.

**2.** Werner A, Freesmeyer M, Kühnel C, Drescher R, Greiser J. Liver PET Reloaded: Automated Synthesis of [68Ga] Ga-BP-IDA for Positron Imaging of the Hepatobiliary Function and First Clinical Experience. *Diagnostics.* 2023;13:1144.

**3.** Freesmeyer M, Kuehnel C, Opfermann T, et al. The use of ostrich eggs for in ovo research: Making preclinical imaging research affordable and available. *Journal of Nuclear Medicine.* 2018;59:1901-1906.

**4.** Ekman M, Fjälling M, Friman S, Carlson S, Volkmann R. Liver uptake function measured by IODIDA clearance rate in liver transplant patients and healthy volunteers. *Nuclear medicine communications.* 1996;17:235-242.

**Stability of [^68^Ga]Ga-BP-IDA in-vitro**

[^68^Ga]Ga-BP-IDA main peak: 8.4 – 8.6 min.

**
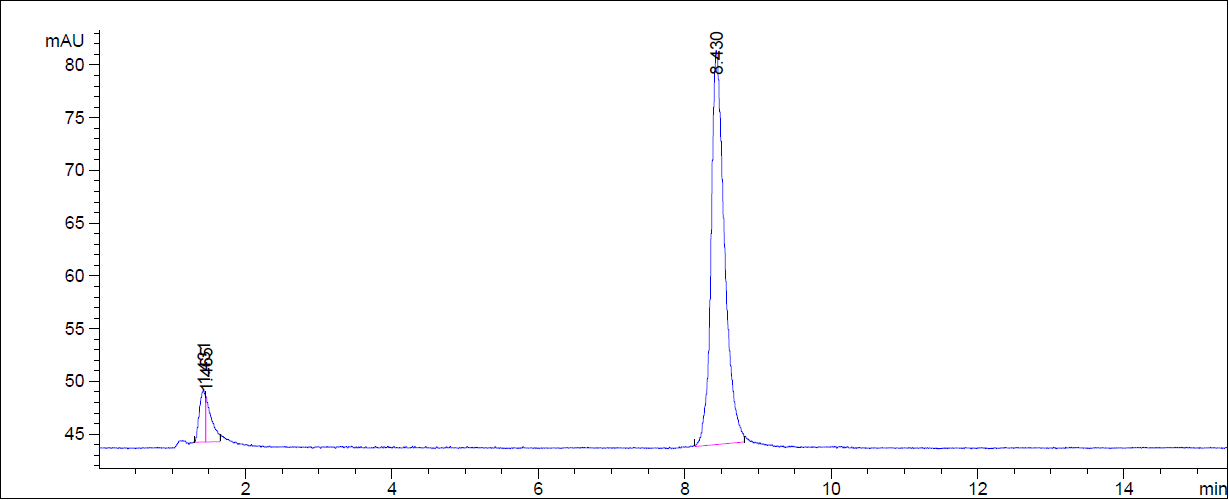

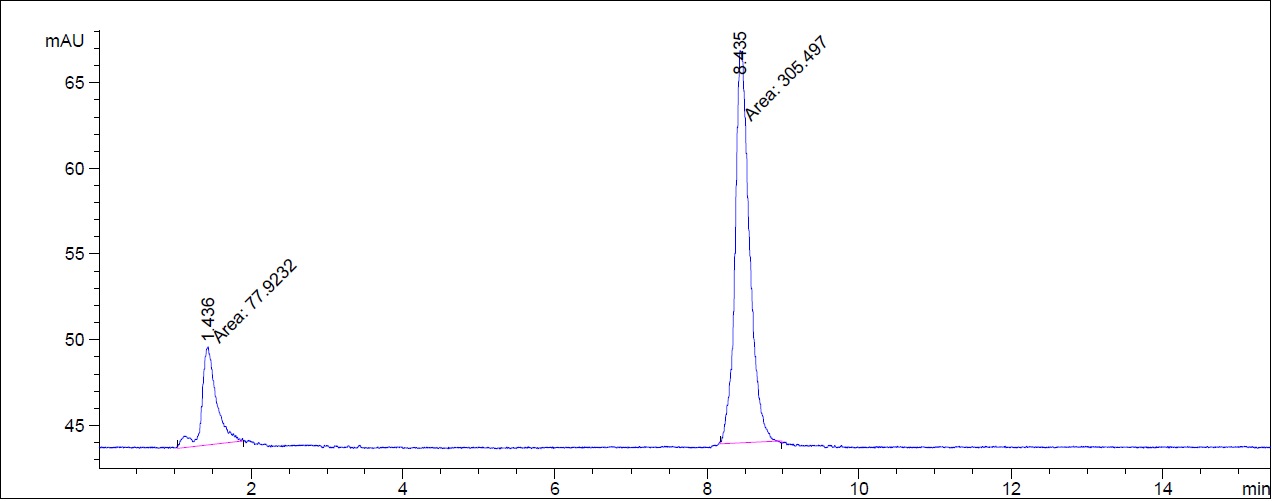
**

Human Serum, 37°C, 80 min

RCP = 79.7%

Human Serum, 37°C, 45 min

RCP = 90.3%

**Fig. S1**. Radio-HPLC chromatogram of [^68^Ga]Ga-BP-IDA incubated in human serum at 37 °C.

**
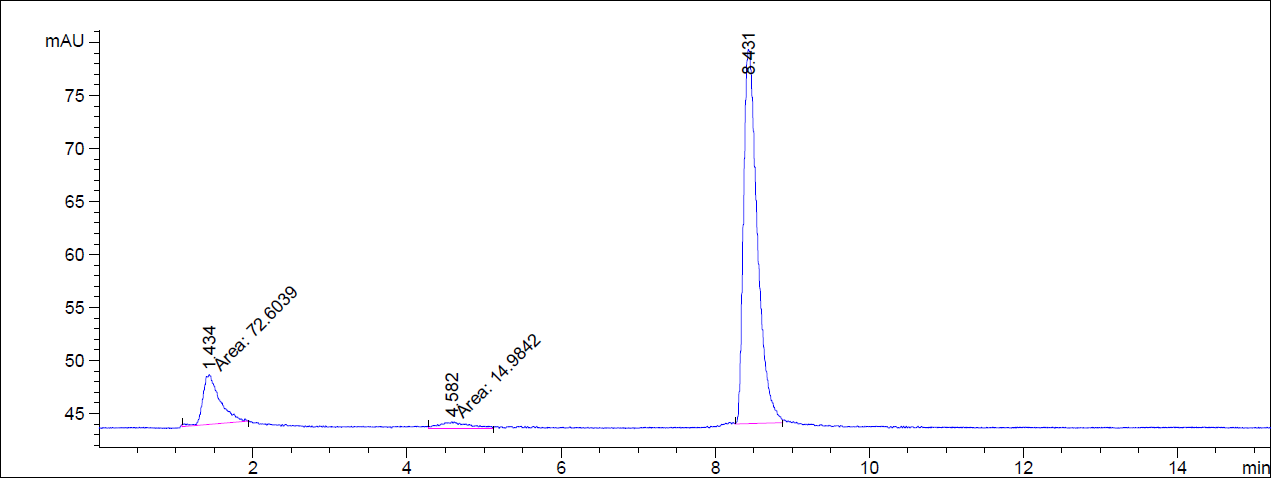

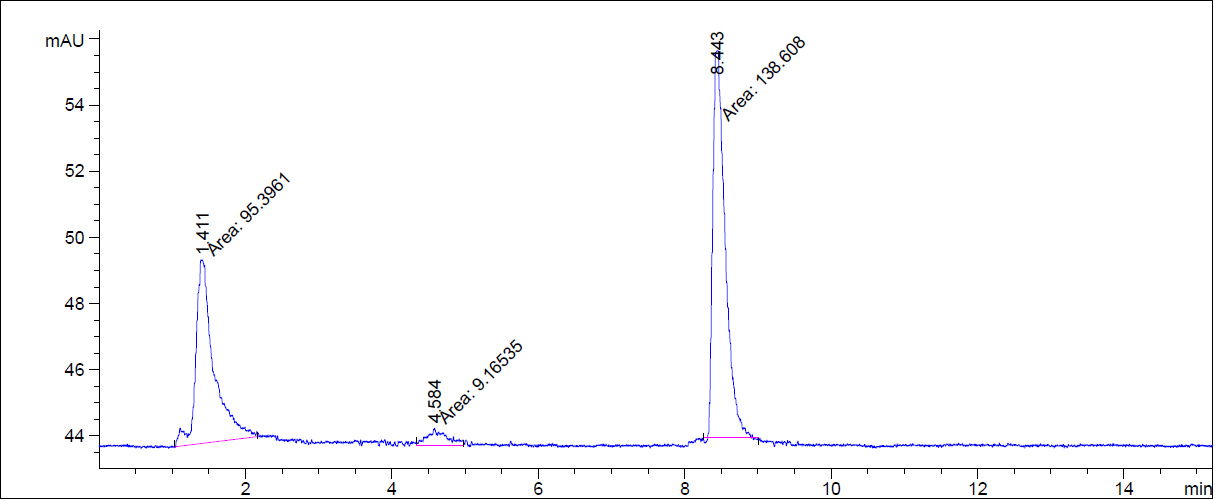
**

PBS, 25°C, 25 min

RCP = 83.5%

PBS, 25°C, 60 min

RCP = 57.0%

**Fig. S2**. Radio-HPLC chromatogram of [^68^Ga]Ga-BP-IDA diluted with PBS solution at room temperature.

**
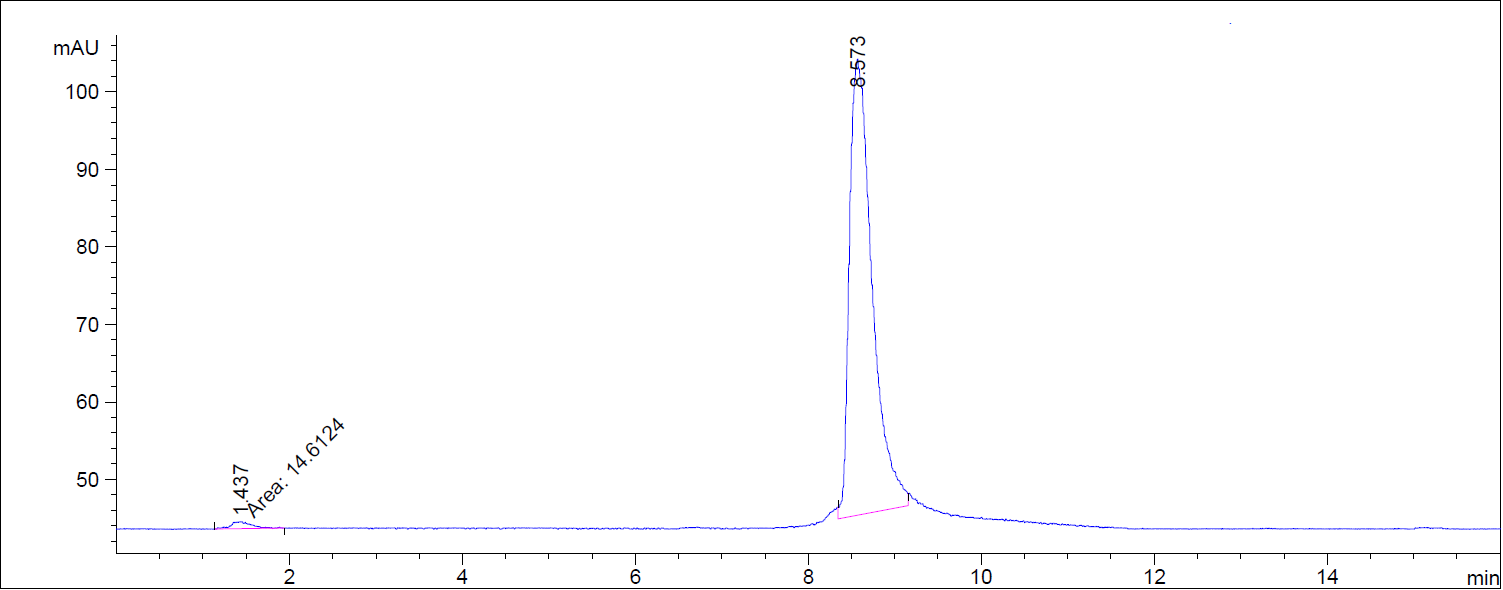

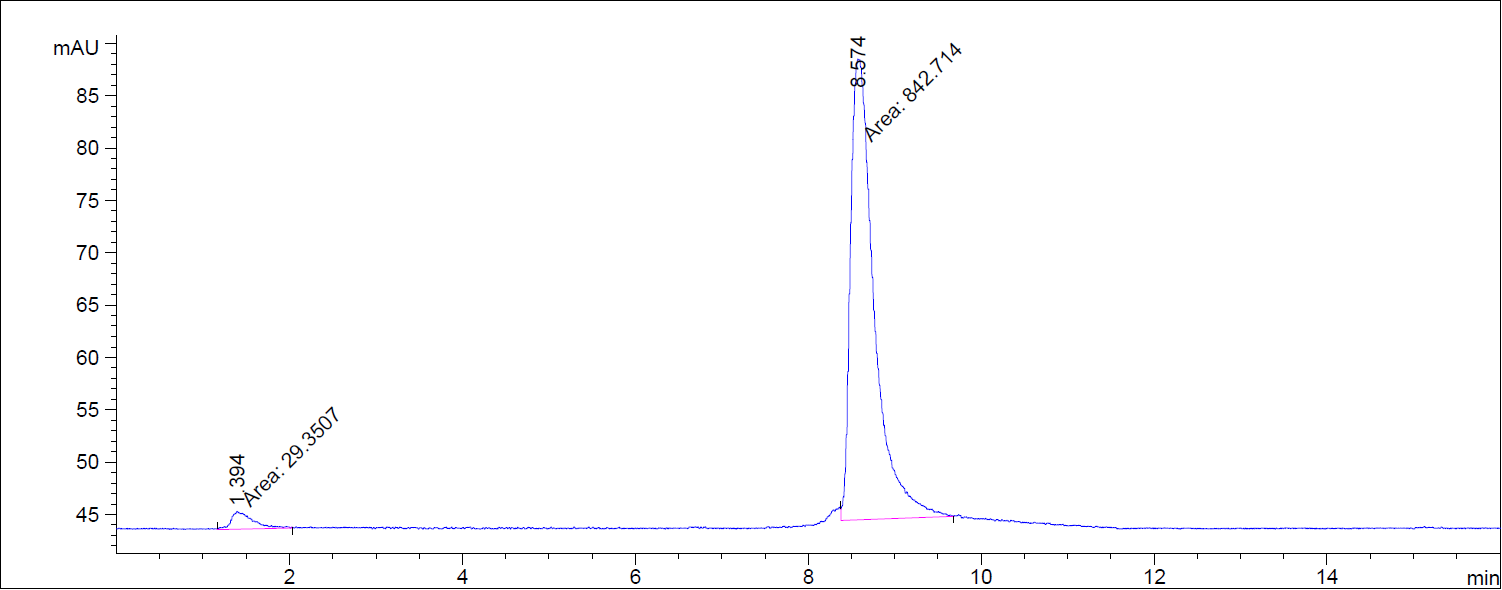

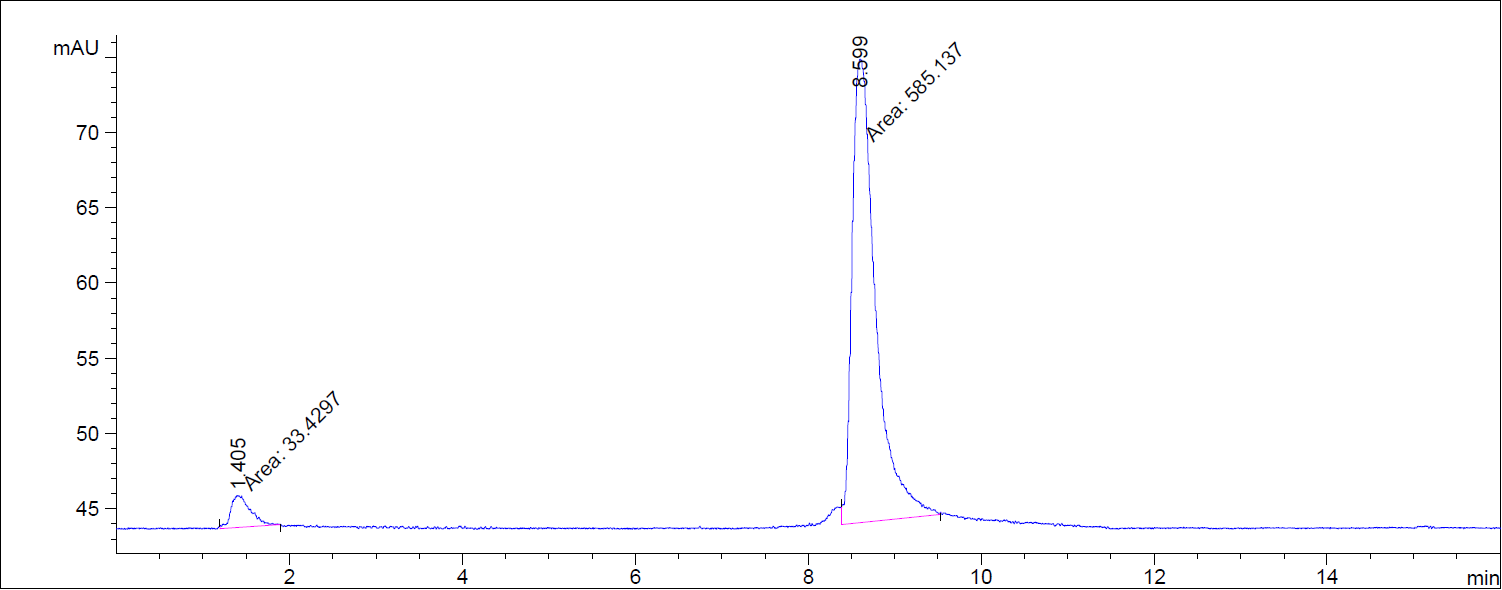
**

Saline, 25°C, 45 min

RCP = 94.6%

Saline, 25°C, 15 min

RCP = 98.7%

Saline, 25°C, 35 min

RCP = 96.6%

**Fig. S3**. Radio-HPLC chromatogram of [^68^Ga]Ga-BP-IDA diluted with saline at room temperature.

**Metabolite analysis of [^68^Ga]Ga-BP-IDA in-vivo (model: ostrich embryo)**

[^68^Ga]Ga-BP-IDA main peak: 8.4 – 8.6 min.

**
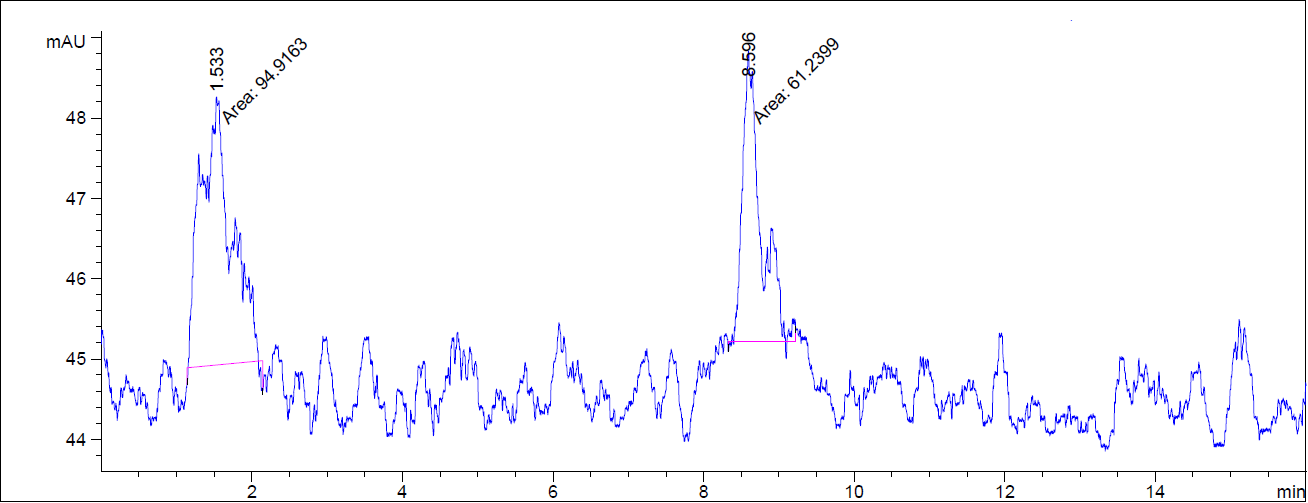
**

blood, 60 min p.i.

**
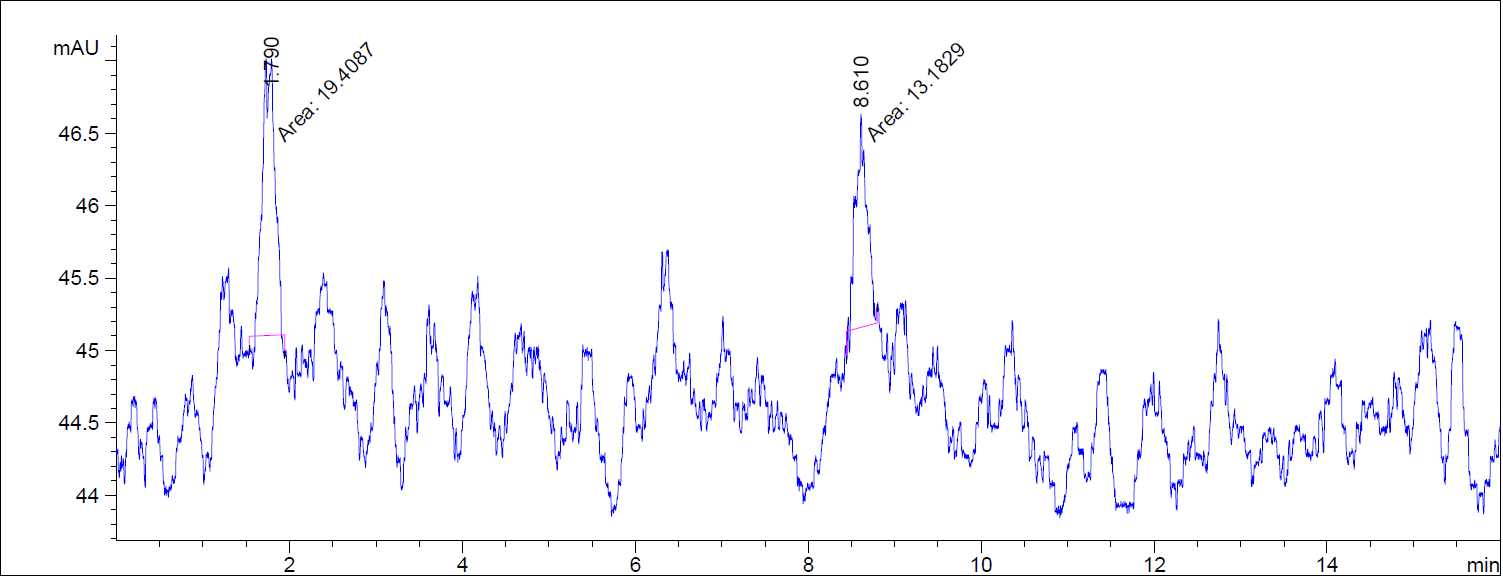
**

kidney homogenate in acetonitrile, 60 min p.i.

**
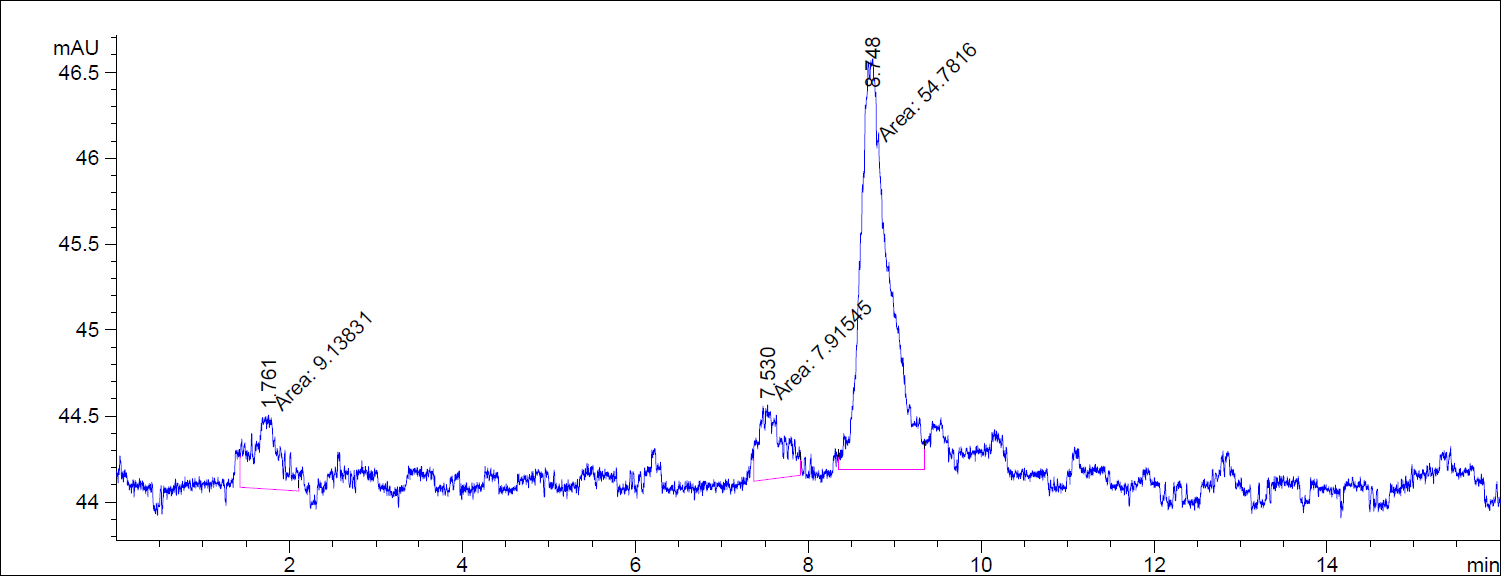
**

liver homogenate in acetonitrile, 60 min p.i.

**
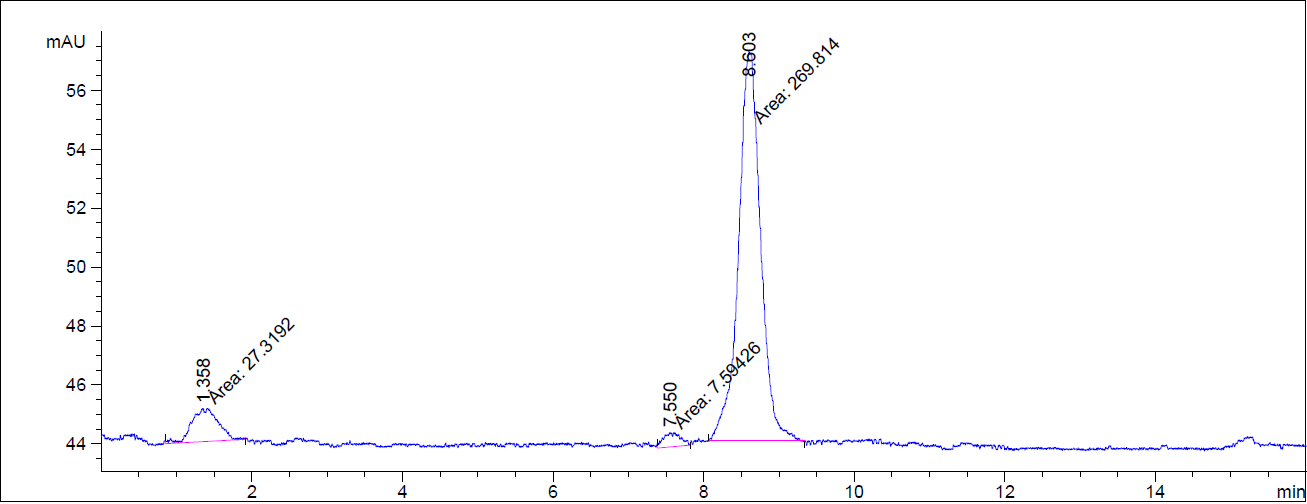
**

stomach content, 60 min p.i.

**
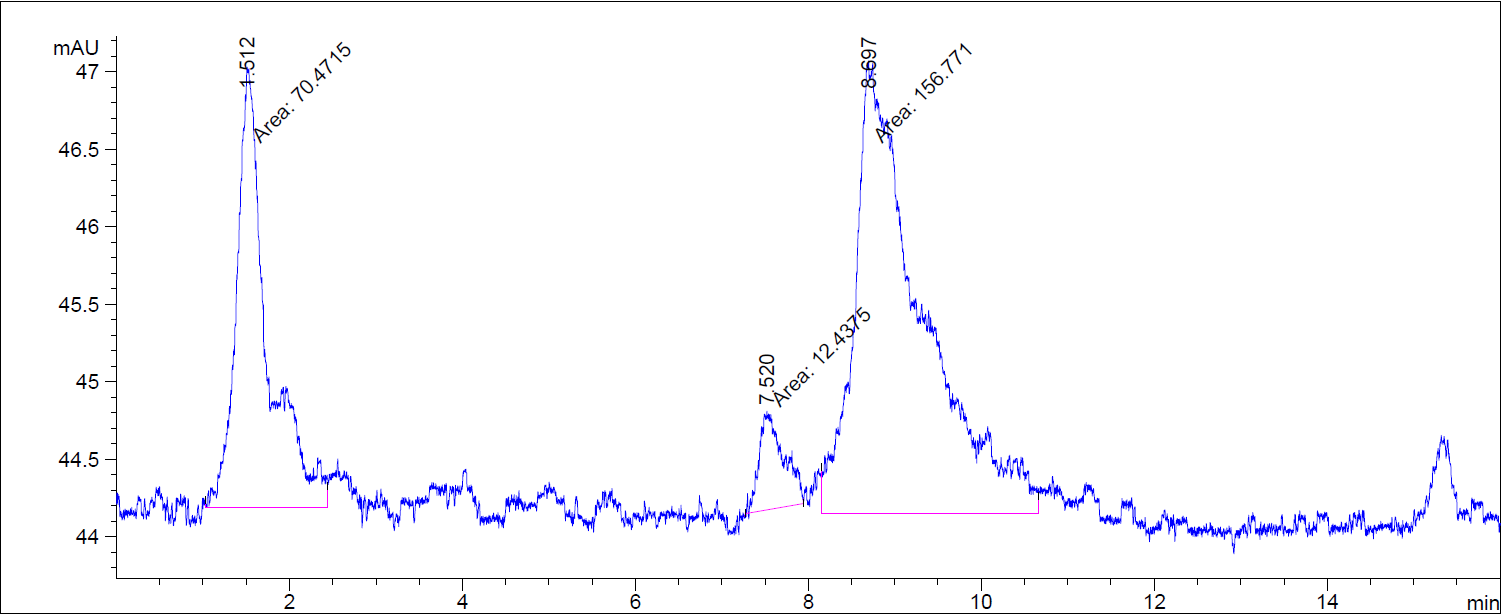

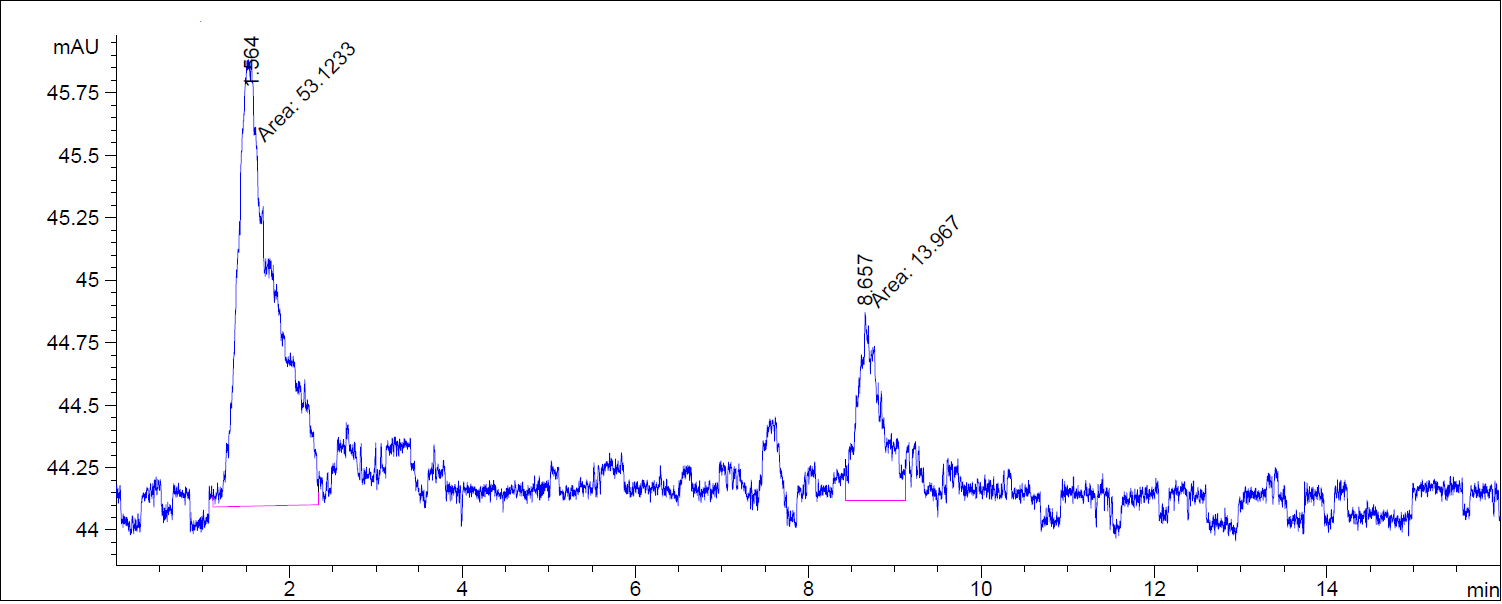
**

intestine content, 60 min p.i.

intestine content, 60 min p.i.,

reinjected 90 min after first sample

**Fig. S4**. Radio-HPLC chromatograms of metabolite probes taken 60 min after injection of [^68^Ga]Ga‑BP-IDA into ostrich embryos.


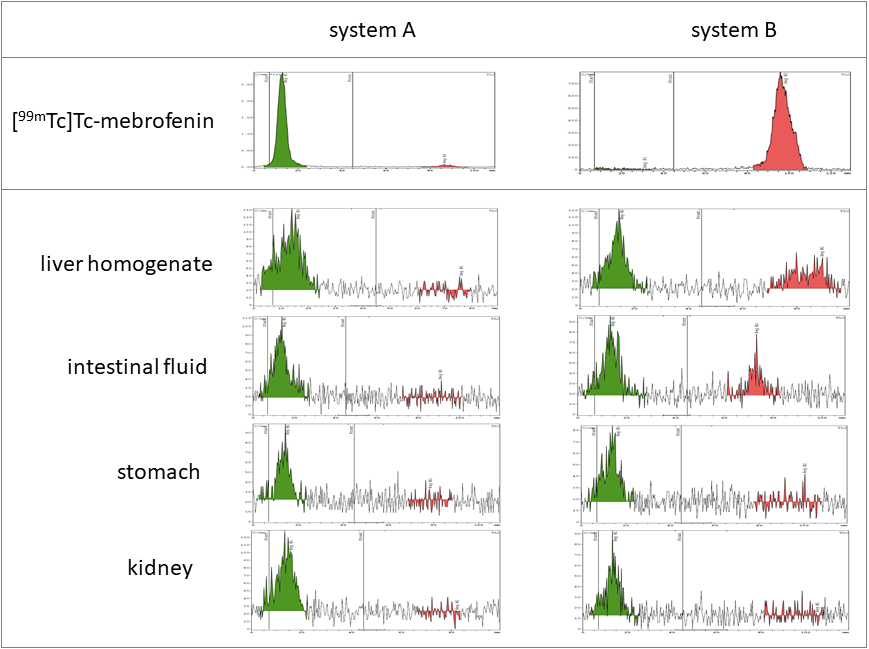


**Fig. S5**. Radio-TLC analysis of [^99m^Tc]Tc-mebrofenin (top) and of tissue samples from ostrich embryos gained 60 min after injection of [^99m^Tc]Tc-mebrofenin.
